# An Inclusive Re-Engagement with our Nonhuman Animal Kin: Considering Human Interrelationships with Nonhuman Animals

**DOI:** 10.3390/ani1010040

**Published:** 2010-12-14

**Authors:** Rod Bennison

**Affiliations:** Honorary Fellow, Macquarie University, Sydney, Australia; E-Mails: ozrodoz@gmail.com; mindinganimals@gmail.com; Tel.: +61-414914040

**Keywords:** animal, nonhuman animal, ecological, inclusion

## Abstract

**Simple Summary:**

The impact that the divide between human and nonhuman animals, between nature and culture, actually has on the planet, on nonhuman animals, cannot be underestimated. The divide is a socially constructed separation, an ‘othering’, of all life on the planet, and has been imposed and systematically used by humanity in order to exploit nonhuman animals and utilise the environment without little thought as to the ramifications. The paper examines such complex questions as ‘what is nature?’, ‘what is an animal?’ and ‘what does it mean to be human?’ Issues considered include species concepts, nature culture dualisms, and the human place within animality. What is recommended is an an urgent need for the adoption of an ethic of ecological inclusion.

**Abstract:**

As humans increasingly acknowledge the effects that they are having on the planet, there is a realisation implicit in these effects that human interrelationships with nature are actually arbitrated and expedited exploitatively. Understanding how the different discourses and histories through which the interrelationships with nature are mediated and actually told and then retold is fundamental to appreciating how humans may relate with nature less exploitatively and in ways that are more inclusionary, particularly with nonhuman animals. Humans perceive nature and individual nonhuman animals in various ways. This paper provides an investigation of how humans have socially constructed nature and their place as either within or outside of it. Such constructions are elaborated conceptually and through narrative. More pertinently, this paper examines how nature and nonhuman animals are perceived and placed within those narratives that humans construct from reality. It is stressed here that such constructions have, and may continue, to lead to a worsening of the effects that humans have on the planet if there is no acceptance or recognition that certain realities exist beyond the exploitative bounds of any human-inspired concept or narrative. This paper therefore provides the groundwork for the foundations of an ethic that is both socially and ecologically inclusive and is based on a soft realist approach.

## 1. The Divide between Human and Nonhuman Animals

The impact of the divide that exists between human and nonhuman animals, between nature and culture, has on nonhuman animals cannot be underestimated. The divide is in fact a socially constructed separation, an ‘othering’, of life on the planet, and has been imposed and systematically used by humanity in order to exploit nonhuman animals and utilise the environment without little thought as to the ramifications.

The effects that humans, particularly those of industrial society, have had on the environment are so immense that there is “an important sense in which it is correct to speak of ‘nature’ as itself a cultural product or construction” [1]. The interrelationships are in large measure impacted by the way that humans, and only humans, ontologically separate themselves from nonhuman animals, and how humans conceptualise their interrelationships with other beings, or the way that humans construct narratives of their life stories [2]. Peterson [3] argues that such narratives may be described more accurately as how humans construe nature.

The separation between human and nonhuman animals is the subject of a long history that extends back before the advent of the Neolithic, and ranges from mythic to modern scientific accounts. What all histories exemplify is “as much about how the narrators view their own humanity as they do about their attitudes and relations to nonhuman animals” [2].

The interrelationships that have, and do exist, between human and nonhuman animals, and between human groups, have varied greatly over time and between cultures. The interrelationships have been, and are contingent upon, the development and structure of the conceptual frameworks and narratives so constructed. What are also most important are not just the actual constructs, but the identity and worth that any individual ascribes to nature in a given context.

## 2. Social Constructivist and Realist Accounts of Nature

Social construction as elaborated here follows Soulé and Lease, and is taken to be any “action based on the ideological persuasion that reality is the product of social interactions and dynamics” [4] where different realities are contingent upon the many cultural contexts that exist across the planet. The social construction of nature has been defined extensively by authors such as Demeritt [5], Hacking [6] and Peterson [3]. Demeritt follows Hacking and distinguishes construction as refutation (deconstruction) or as philosophical critique (phenomenological constructionism, sociology of scientific knowledge, discursive constructionisms and embodied practice, the latter including actor network theory, rhizomatics and hybridity).

Arguing from a realist perspective, Lease boldly claims that “our many representations of nature and human are … always and ultimately failures” [7]. As far as the many examples of supposed human superiority over so-called nature are concerned, he may be correct, but who is to say that a particular representation is not real or truthful. In this regards, Demerrit [8] notes that reality is only ever realised as an artefact of a representation.

Throughout their evolution, humans have increasingly sought to dominate nature in order to fully exploit it. At the same time, humans have invented ways to justify such domination. Humans have done this by seeking to give meanings to nature and culture that can be used to justify exploitation (for example, by economic rationalists on behalf of conservative governments or by consultants on behalf of industry).

The human desire to identify other beings and things as objects, to construct and compartmentalise knowledge and, as such, to search for and ascribe meanings to nature (as well as to animality and humanity), perhaps paradoxically, has been used by humanity to justify their wanton planet-wide destructive ways. By and large, humans, in seeking to find meaning to life and their ‘place’ in the world, see themselves as subjects or members of a separate and definable culture, which is distinct from nature. Nature on the other hand is seen as being made up of all other nonhuman animals, plants, fungi, rocks and so on, which are seen as lesser objects. In excluding themselves from nature, humans have falsely come to see themselves as greater than nature and above it. This has exacerbated the exploitation of nature.

Realist and social constructionist accounts of nature seek to examine why the interrelationships that exist manifest themselves in ways that have become exploitative, and almost entirely exclusionary. Realists and constructionists view nature and the distinctions that humans draw in various ways. Realists view nature as that which exists no matter what the particular representation, whereas constructivists consider nature to be “in both discourse and practice … as socially made, not ontologically given” [9].

Social constructionists, like realists, may be concerned for the effects of humanity on nature and the need for the protection of the environment, but their argument, which is “preoccupied with the mechanisms and outcomes of construction” [9], detracts from the ‘reality’ of the issue. That reality is that a real problem exists with the human exploitation of nature, and no end of argument as to how humans construct or construe their world will alleviate the exploitation that otherwise exists.

A concept that is both socially and ecologically inclusive provides an alternative focus, and particularly not on the rightness of a competing argument, or with an overabundance of constructed meanings that can be ascribed to human dealings with nature. The focus of an inclusive framework is positioned upon the actual place and recognition of all beings as individual subjects within a narrative or framework that otherwise exists, whether within or indeed beyond, the bounds of human perception, and as such, within the ecological whole or biological community. In this, it is recognised that all constructs are given form from the many pieces of information that humans utilise in their deriving their own view of reality, that is, from perception.

## 3. Definitions of Nature

Nature is one of the most fraught words in the human vocabulary, let alone concepts in the human psyche, and the many meanings ascribed to it involve an extraordinary long history in the development of ideas concerned with idealism, God, governance, society, the environment, plants, nonhuman animals, and more. As Soper claims, its “complexity is concealed by its use in a wide variety of contexts” [1]. In this regard, the phenomenological constructionists, Macnaghten and Urry, note that it is the “abstraction of a singular nature from the multiplicity of lived experiences (starting over two thousand years ago) that was to prove so critical for subsequent human responses to the physical world” [10], and changing human responses to the natural world have been elaborated extensively by many authors.

If every human were to ascribe a meaning to what they each perceive nature to be, each would arrive at a plethora of different meanings that bare little resemblance to what one was indeed searching. All humans conceptualise and give form to what they see nature to be in distinct ways. However, nature exists beyond the bounds of any individual human perception or construction. Not all accounts are necessarily correct. For example, most if not all humans may recognise the sky to be blue; there is a scientific reason why it is so. There may be general agreement on such a case, but the reasons why each human ascribes to the sky a perceived blueness beyond the scientific reality may be different.

Williams, in his observation that the word ‘nature’ “is perhaps the most complex in the English language”, defines three meanings of the word, as the ‘essential quality’ of something, the ‘inherent force’ that directs the world, humans or both, or the ‘external, material world’ [11]. Similarly, Ellen suggests that humans have a propensity to identify things or natural kinds, to see nature as something ‘out there’, or what is not in ourselves and what emerges when we attempt to essentialise an unknown phenomenon [12].

Further, Castree [13] groups nature into three broad categories of meaning which, in the main, follow Ellen, where nature can be seen as the essence of something, as an area external to and unaltered by humanity, or as the entire physical world of which humans are a part. The latter points in Castree's definition forms the basis of the argument of this paper; that nature is the entire physical world inclusive of humanity and human culture.

On face value, one would ‘naturally’ be inclined to accept such categorisations as described above, but are inadequate as such definitions do not recognise that nature cannot be categorised. To do so is to fail to recognise that the ‘essential quality’ of something, the ‘inherent force’ that directs the world and the ‘external, material world’ is the the same thing. Nature is that word; the concept that humans have devised to describe that which they cannot. Essentially, nature ‘is’. Or, nature is all.

To be more inclusive or holistic we should delve more fully into the metaphysical and spiritual; at least into our inner selves. Not to do so is to fail to recognise our place within nature. In this regard, Descola notes more pertinently and accurately, a further three ways of constructing nature and the boundaries that humans set between themselves and nonhuman animals, between self and otherness. He outlines these concepts as ‘totemism’, ‘animism’ and ‘naturalism’; defining ‘totemism’ as where humans use “empirically observable discontinuities” between and to delimit ‘species’, where nonhuman animals are treated as signs, and ‘animism’ as a “symmetrical inversion of totemic classifications”, where nonhuman animals are treated as the terms of a relation or endowed with “human dispositions and social attributes” [14].

Naturalism, on the other hand, is where nonhuman animals are treated as ‘other’ to human culture. Naturalism is “simply the belief that nature does exist, that certain things owe their existence and development to a principle extraneous both to chance and to the effects of human will” [14]. With regard to the third point, Peterson reminds us that such a construct is the “dominant construction of nature in modern Western cultures … (and) not the only way to construct it” [3]. Naturalism provides an ontological domain where “nothing happens without a reason or cause, whether originating in God” or “immanent to the fabric of the world”.

Seeing nonhuman animals as totems, or in an animist sense, has more often than not directed anthropologists, amongst others, to relate such constructions of nature as belonging to ‘primitive’ cultures, predominately distinguished as societies or cultures of hunters and gatherers. Ingold asserts that the “ontological dualism … of which the dichotomy of nature and culture is the prototypical” is rejected by such cultures [15].

It could be argued that the questioning of nature as something that is seen as transfixed in place, space and time, yet something that we cannot adequately define, let alone categorise or socially construct, is itself a fallacious enterprise. Many social anthropologists, human geographers and sociologists that use social constructionist approaches, including Demerrit [5,16], Entrikin [17], Murdoch [18], Macnaghten and Urry [10], and Proctor [19,20] fall into this trap. For example, Philo and Wilbert, in trying to conceptually place nonhuman animals to assist in the purview of their field of animal geography, incorrectly seem to consider only vertebrates to be animals [21]. Reality seems to be too difficult for some to consider.

Unlike these authors, Soulé, arguing from a realist perspective, provides an accessible analysis of nature, and which in practice may allow incremental steps to be made towards inclusiveness [22]. He argues that every human ascribes several similar conceptual layers to what they each perceive nature to be [22]. The list that he defines form nine distinct cognitive formations from a non-exhaustive list of ‘natures’, which extend from the most scientific, exploitative and “bullying” to a formation which he names as ‘Magna Mater’, the “hunter-gatherer, animistic, pagan sense of divine oneness or monism” [22]. The latter cognitive formation is similar to the animist definition of nature given by Descola [14], not least the many accounts provided by late 20th century environmentalists and animal protectionists.

Importantly, Soulé claims that all such cognitive formations of nature are indicative of the “polymorphic, fragmented nature of human occupations and preoccupations in a civilisation that encompasses an extraordinary range of subcultures, levels of affluence, contact with natural habitats, and philosophical sophistication” [22]. This idea is indicative of the problems associated with the enterprise of defining nature even by realists, and the very ‘naturalness’ of the term itself. It is more likely that all conceptual layers outlined by Soulé are in fact sub-categories of his ‘Magna Mater’ formation, so no matter what perceptions of nature are held by an individual, those different perceptions represent various integrated layers of an overarching divine oneness with nature.

Soulé and Lease [4], and others, who argue the point from a realist perspective may well indeed go a long way towards inclusiveness when they challenge the validity of the arguments posed by those who seek to construct definitions of nature, that as such, lie beyond the bounds of reality. The reality is that there is but one nature, albeit perceived by every individual human animal, and probably many nonhuman animals for that matter, in ways that that individual wants it to be or is able to perceive. There is a growing list of scholars that ascribe to a similar realist position including Crist [23], Kidner [24], McKibben [25], Peterson [3] and Soper [1], as well as those arguing for a more Marxist position like Benton [26] and Noske [27].

The position of these authors stand in stark contrast to the arguments posed by social constructionists who, like Macnaghten and Urry, seek to demonstrate that there is actually not one single ‘nature’, but a “diversity of contested natures … each constituted through a variety of processes from which (they) cannot be plausibly separated” [10]. One can only question the very rationality of how we can distinguish such natures if they cannot be separated in the first place.

Stretching the point, one could even complicate the problems that do exist by elaborating a position that nature may not even exist at all (that in itself would be a construct!). Dwyer states that nature as “westerners know it is an invention, an artefact” [28]. Peterson alerts us to the many dangers of such nihilistic constructionist accounts when she states that such perspectives assert that humans “live in culture, which includes our ideas about nature, and there is no nature apart from those ideas” [3]. Peterson asserts that an “awareness that reality is socially constituted is crucial because it guards against the danger of reifying human constructs” [3]. Strong versions of the social constructionist argument are ethically problematic and politically loaded. Generally, however, social constructionists merely elaborate a position whereby the environment, that is nature, is seen by human animals as a distinct and “real entity, which is … substantially separate from social practices and human experience” [10].

The major failure with this narrow approach is that it is at the expense of individual perception, a point reinforced by those claims made by Soulé [22], that nature is what individual humans perceive it to be (no matter how many conceptual layers are recognised) as well as what humans collectively see it as in its totality. Social constructionism, if not in intent, is an approach that seeks to reinforce a separation of humanity and culture from broader nature, subject from object.

Social construction is in fact dangerous as it supports a position of our continued and increasing exclusion from nature, and of nonhuman animals in particular. Indeed, the more we reflect on any matter, the more likely that we construct barriers; this in turn would, if this be the case, further alienate humans from nature. Soper notes that in this regard, that it was Heidegger who, “comes close to suggesting that we are (actually) alienated from the world in the very act of cognitively reflecting upon it” [1].

Given that environmental despoliation and abuses of nonhuman animal continue to be perpetrated by humans, it should be recognised that the ‘entire’ environment, including both nonhuman and human animals, may very well be seen by some as an entity separate or detached from our purview. It should never be open to abuse, as may occur if the constructionist enterprise and exclusionary worldviews gain further currency.

## 4. The Dualism of Culture and Nature

Soper argues from a soft realist perspective that:

nature is that which humanity finds itself within, and to which in some sense it belongs, but also that which it also seems excluded in the very moment in which it reflects upon either its otherness or its belongingness [1].

However, one would indeed question why most humans have otherwise considered themselves in a state of being that excludes them from the entire ‘natural’ environment. Culture, that state of being, has, like nature, been conceptualised as something that stands as separate and above nature and in ways that has been ultimately detrimental to human and nonhuman animal alike. The dualism between nature and culture has been a major component in the advance of Western civilisation “at the expense of nature” [29].

MacCormack suggests that culture is that “which is arbitrary and artificial” whereas nature is that “which is innate in our primal heritage” [30]. In the same volume, Strathern spuriously claims that “there is no such thing as nature or culture … no consistent dichotomy, only a matrix of contrasts” [31]. This also denies the fact that ‘human’ culture is a phenomenon of recent Pleistocene origins. Monaghan and Just defined culture as “shared patterns of learned behaviour” and has to do with “those aspects of human cognition and activity that are derived from what we learn” [32], whilst Alvard defines culture as merely “socially transmitted information” [33], no matter what the group of animals. It is not the main purpose of this paper to define culture; however, as culture has been elevated to a position by the more dominant human societies to be supposedly ‘above’ nature, it is important to at least reflect upon the separation that exists between culture and nature so that a case for inclusion can be elucidated.

It has been recognised that the separation that exists between nature and culture is in large part a reflection of the actions and exploitative tendencies of dominant or colonising societies. In this regard, the question as to whether humans reside inside or outside nature has often been linked to whether a social group or ‘culture’ has reached a particular technological or cultural hierarchical level [5]. Whereas, hunter-gatherer societies are often represented as living in a ‘lower’ state of supposed ‘savagery’ subject to the universal laws of nature, “modern people are imagined as having escaped these biological imperatives” [5]. Such dualistic notions are not only reflective of a racialised and gendered stereotyping which has been used to justify an oppression and dispossession of most, if not all ‘primitive’ (that is, non-Western) societies, women and sexual minorities, but is also used to legitimise the exploitation of nonhuman animals. Oppression and dispossession continue to be part of the Western condition and symbolic of the conditions those in the West place on nature, which is “most obviously recognisable as what is ‘out there’, what is not ourselves, and ‘that which can take care of itself’” [12].

The dualism of nature and culture has been created through the construction of experience and is “*in nature* only so far as *Homo sapiens* is also in nature… culture emerges from nature as the symbolic representation” of humanity within it [12]. However, as many authors have indicated, the disengagement of culture from nature, humanity from animality, is what lies at the “root of our ecologic crisis … and behoves us to reverse the order of priority” [2].

The threats to socio-ecological well-being was recognised in the West at least as far back as George Perkins Marsh in 1864, but it was not until the likes of Aldo Leopold, Rachel Carson and Lynn White Jnr. in the 20th Century, that the threats posed to the ecological foundations of life on Earth was most vociferously asserted. In responding to White's famous charge, Moncrief strongly contends that the cultural basis for the ecologic crisis lies with “the forces of democracy, technology, urbanisation, increasing individual wealth, and an aggressive attitude toward nature” [34].

Ellen stipulates that “the emergence of culture necessitated a bifurcation between experience and representation, and all cognition ultimately derives from experience. In the most fundamental sense, culture is the symbolic representation of experience, and evolution has effected an a priori dualism with our dealings with the world” [12]. Human civilisation would thus appear pre-disposed to dualism. But surely, culture exists only in so far as our own existence allows it to be so, that is, through cognitive realisation and perception? In this sense, nature (the environment made up of humans, nonhumans, plants, rocks, and so on) exists; with ‘human’ culture existing within it entirely, realised and actuated through evolutionary processes, and that it is ‘human’ culture that has been, and can only be, constructed.

Nevertheless, not all human societies have alienated themselves from the environment as far as have ‘Western societies’. As stated above, so-called ‘hunter-gatherer’ societies are often seen as existing in nature, a particularly pertinent point as regards the need for a new inter-relationship between human and nonhuman animals. Unlike Western societies that rely on dualistic assumptions such as nature existing apart from humanity, many Indigenous societies “see no essential difference between the ways one relates to humans and to nonhuman constituents of the environment … humanity and nature merge into a single field of relationships” [2].

As Knudtson and Suzuki note regarding the worldview of the Tukano Indians of Amazonian Colombia, “the natural world (including humans) is rooted in a vast web of reciprocal relationships that have always existed between all elements in nature” [35]. Moreover, as Descola notes of the Tukano, “reciprocity is based on a principle of strict equivalence between humans and nonhumans sharing the biosphere” [14]. Such non-dualistic narratives, amongst the many from societies of First Peoples, may help 21st century people to be more ecologically inclusive, and relegate objectivism to the traditions of Western scientific enquiry.

## 5. Nonhuman Animal Culture

Scientists continue to look for, or objectify, culture in nonhuman animals. To date, many scientists have attempted to show that cultural expression within nonhumans is confined to single behaviour patterns, such as song-bird dialects [36] and feeding techniques used by rats [37]. However, some nonhuman animals have been shown to possess a much broader range of cultural traits. Of particular importance is the existence of culture in other great apes [38], and as is evident in their language and speech [39,40]. Rendell and Whitehead have also provided evidence of cultural traits in some whales and dolphins [41], and Grant and Grant have provided evidence of cultural inheritance in Darwin finches [42].

Nevertheless, such evidence again begs the question of ‘what is culture’? The evidence seems incontrovertible that culture is that which is derived through learned social behaviour. Laland and Hoppit have claimed that culture is evident in “those group-typical behaviour patterns shared by members of a community that rely on socially learned and transmitted information” [43].

Both Tomasello [44] and Alvard [33] consider that it is cumulative cultural adaptation that distinguishes the human from nonhuman animals, suggesting that the human has a form of culture distinct from other animals only in degree. The complex use of language and the vast array of artefacts, technologies and narratives, are those specific cultural traditions that make humans distinct, but, importantly, not dichotomous from their nonhuman animal kin. Such traditions do not distinguish human animals apart from nature, but a part of it, albeit distinct from nonhuman animals.

The question therefore becomes not whether other animals possess culture; but whether any particular group of animals have acquired a tradition through learned behaviour or social interaction that can be considered a cultural trait. Culture is, thus, merely an expression of natural evolutionary processes and indicative of particular animal societies, if not all animal groups. At the very least, therefore, any animal that interacts in a cultural setting must, first and foremost, be self aware and a possible subject of moral agency.

To recognise the existence of culture is to recognise the existence of a particular trait of a particular group of animals, be they human, bonobo or killer whale. However, we are more than just recognising shared or unique characteristics; by doing so we are actually repositioning ourselves within the environment (that is, being inclusive) and acknowledging our animality, and in certain instances the humanity or personhood of some other nonhuman animals. So, if some, if not all, nonhuman animals possess culture or personhood, and if humanity is a sub-set of animality, then what is an animal, but more, what does it mean to be human?

## 6. Humanity, Animality, Personhood and the ‘Species’ Concept

In recognising our humanity there is an implicit, if largely unrecognised, admission of our animality. Much ethnographic, anthropological, biological and genetic work over the past thirty years has resulted in increasing admissions of the interconnectedness between all animals, human and nonhuman animals alike. Ingold outlines the associations that humans have developed with nonhuman animals in their shared histories, and argues that there is a “strong emotional undercurrent to our ideas about animality”, and to analyse those ideas exposes “highly sensitive and largely unexplored aspects of the understanding of our own humanity” [45]. This is particularly true considering that the very notion of human animality threatens human perceived superiority over nature and the otherwise wilful human exploitation of nonhuman life, highlighted especially by those distinctions that human animals have formerly drawn between themselves and nonhuman animals to justify exclusionary and exploitative practices.

There are many ways in which the term ‘animal’ has been defined and, in the main, concern the perceived boundaries between human and nonhuman animals, and between forms of the living and the nonliving. The most basic definition of ‘animal’ lies in the distinctions that can be drawn between those entities that have the ability to either move or autonomously interact, compared to those that do not [46]. The common distinction drawn is that between taxonomically distinct entities, namely animals as compared to plants and fungi, which Sebeok denotes respectively as ingestors, producers and decomposers [47].

Zwart uses three historical philosophical positions to define all animals. Zwart notes the Cartesian notion of animals as machines, the Kantian notion of animals as distinct organisms, and the Heideggerian argument that nonhuman animals, as much as humans, have a way of “dwelling” in the world, that “to live is not a characteristic of living beings, but rather their basic way of being” [48]. This latter definition is particularly important given Clark's concerns regarding the boundaries of the human moral community, as described below.

Scientific systems of classification are the most common way in which humans construct distinctions between themselves and nonhuman animals, not least to define their own humanity being as distinct from a unique and separate animality. There are many classificatory systems that have been applied by humans, from the Aristotelian system of ancient Greece which dominated Western thought up to and including the Scientific Revolution, to the Linnean and variant taxonomic systems of modern science, to now less common folk taxonomies used mainly by First Peoples for millennia.

Early scientific classificatory systems tended to be used to explain adaptive change and perceived differences within human groups and between nonhuman animals. Importantly, such differences were integral to the notions of continuity and plenitude, and the concept of the *Great Chain of Being*. Classificatory systems were eventually used to advance evolutionary theory at least from the 1860s, until the application of cladistics, which stressed the importance of describing anatomical and physiognomic characteristics, before the elucidation of evolutionary connections.

More recently, genetic coding and more recent studies in animal morphology, has clarified many of the relationships that exist between human and nonhuman animals. At the same time, the application of genetics has disrupted the utilisation of the human constructions of genera and species which really began to break down in the late 18th century when adaptive change was recognised and evolutionary science began to be formulated.

Be that as it may, no matter what genetic relationships exist, all classificatory systems are social constructions; as John Locke noted as early as the 17th century, “the boundaries of the species, whereby men sort them, are made by men” [49]. In the early period of the development of modern Western biology and ecology, many natural scientists rejected the concept of species, although they were arguing from the perspective that such constructions do not adequately reflect what (their) God had planned. Georges Louis Leclerc, the Comte de Buffon, said for example:

*il eût parlé plus clairement, & ses divisions eussent été plus varies & moins arbitraires; car en général plus on augmentera le nombre des divisions des productions naturelles, plus on approchera du vrai, puisqu'il n'existe réellement dans la nature que des individus, & que les genres, les ordres & les classes n'existent que dans notre imagination* (author's translation: it's more than clear, as one's divisions become more varied and less arbitrary; because in general the more one increases the number of divisions of natural entities, the more one approaches reality, since in reality only individuals exist in nature, and genera, orders and classes only exist in our imagination) [50].

Traditionally, and more recently, species have been defined as “the units of biodiversity … reproductively isolated natural entities … characterised by unique specific mate recognition systems” [51]. However, distinct scientific definitions should not be seen as so accessible. Mayden, for example, recognises at least 22 decidedly different biological definitions [52], with Mishler noting that the problem of the lack of a truly distinct scientific definition, lying not within what exists in nature, but in the accepted Linnean classificatory system itself [53].

Classificatory systems and ‘species’ as distinct units within those systems are artificial and have come under increasing attack; for example by Dunbar [54], Elstein [55] and, most notably, in the edited volume of Wilson [56]. Dunbar notes ‘species’ are:

matters of convenience rather than biological reality. The real world consists only of individuals who are more or less closely related to each other by virtue of descent from one or more common ancestors [54].

Dunbar even goes far as to say that classificatory systems have been designed to merely satisfy human curiosity and the need to reduce information overload [54].

Lovejoy earlier asserted that classificatory systems are artificial systems which:

tended to give the notion of species a peculiar prominence in scientific thought, to encourage the habit of thinking of organisms, and of other natural objects, as falling into well-differentiated classes, rather than as members of a qualitative continuum [57].

Beyond satisfying curiosity and scientific endeavour, albeit alerting us to the real connections that exist between all animals, classificatory systems merely enmesh humanity in a seemingly superfluous construction of nature that humans use in their exclusion and exploitation of nonhuman animals.

No matter what taxonomic systems elucidate regarding the distinction between animal groups, all individuals (no matter what the relationship) may be linked as descendents of common ancestors, but they are also “potential co-ancestors of a common descendent” [44]. Clark shows that biological ‘species’ are not natural kinds, because when the boundaries of the ‘moral community’ is widely defined, or at least sufficiently enough to include all human descendants, then those same boundaries must encompass *all* nonhuman animal life that the human has descended from [58]. This runs directly counter to the supposed moral superiority of the human animal.

Clark affirms that there may be real or reproductively isolated ‘species’ as any scientific realist account may assert (what Kant called ‘realgattungen’, or collections of interbreeding populations) [58]. However, this should not deny the fact that ‘species’ may also exist and be defined as morphologically similar groups of life forms (what Kant called ‘arten’), as is especially expressed when using a folk taxonomy. Given Clark's fluid approach, how should ‘species’ be otherwise defined if not as a scientific construct of successfully interbreeding or morphologically similar populations?

Clark defines speciation as the development of social, geographical and physiological barriers to successful interbreeding, yet contends that the only natural kinds are indeed not constructed species or organisms, but those individuals with a “shared, distinctive nature” [59]. Similarly, Dawkins sees a ‘species’ in terms of the traits that particular groups of individual life forms share to survive and reproduce [60].

The notion of continuous species or ‘formenkreis’ (literally, ‘ring species’) is probably closer to reality than are constructed scientific taxa [58]; similar to what Sterelny calls ‘phenomenological species’, that is “recognizable, reidentifiable clusters of organism” [61]. Three examples can be drawn to elucidate the notion of formenkreis, which can be seen in real time and across the millennia. The first example is given by Dawkins, who outlines the differences between the Northern Hemisphere populations of the herring gull and the lesser black-backed gull:

In Britain these are clearly distinct species, quite different in colour. Anybody can tell them apart. But if you follow the population of herring gulls westward round the North Pole to North America, then via Alaska across Siberia and back to Europe again, you will notice a curious fact. The ‘herring gulls’ gradually become less and less like herring gulls and more and more like lesser black-backed gulls until it turns out that our European lesser black-backed gulls actually are the other end of a ring that started out as herring gulls. At every stage around the ring, the birds are sufficiently similar to their neighbours to interbreed with them. Until, that is, the ends of the continuum are reached, in Europe. At this point the herring gull and the lesser black-backed gull never interbreed, although they are linked by a continuous series of interbreeding colleagues all the way round the world [62].

A second example can be seen in the differences that exist between the Chihuahua and the Great Dane. These dogs cannot breed successfully, they are not realgattungen, neither are they morphologically similar. But who is to deny that they are not both dogs? They are from the same species, as is provided by the concept of formenkreis.

The most famous example, however, lies closer to home. It can be strongly argued that chimpanzees, bonobos and modern humans, and other hominids, all belong to the same formenkreis. Unlike the above examples, the hominid lineage can be traced across seven million years of earth history. The ‘missing links’, often sought so passionately by anthropologists, are no more than individuals in a ‘chain’. True, the bonobo may not be able to successfully interbreed with modern people, that is they and modern day humans fit the scientific descriptor of realgattungen, but both are directly linked through their descendants as ring species, as much as the Chihuahua and Great Dane are linked to wolves.

Dawkins relates the very close relationship that exists between chimpanzees and modern day humans. He notes that if an individual chimpanzee and an individual human each linked hands with their direct ancestors, and they with theirs, the distance between the individuals and their common ancestor is less than half the distance between the east coast of Africa and the Rift Valley [62]. Such closeness can be seen in the genetic relationship that we both share; in fact, chimpanzees are more closely related to modern day humans than North American red-eyed vireos are to white-eyed vireos (birds) [63].

A similar analogy could be drawn from the possible differences between Flores people (*Homo floresiensis)* and modern peoples. Dependent upon further elucidation of the genetics of Flores people, Flores people may not be able to breed successfully with modern humans alive today. Both modern and Flores people may be realgattungen, but that does not mean that Flores people and modern humans, along with other hominids, such as the australopithecines and the other great apes, are not all members of the one ring species.

Simply, the chimpanzee, or indeed, Flores people are like the Chihuahua or herring gull, while the modern day human is like the Great Dane or lesser black-backed gull. Extending the analogy, chimpanzees, bonobos, Flores people, Neanderthal and modern day peoples may be varieties (‘rassen’) from the same continuous or ring species of African-derived ape. Such apes represent populations of individuals that “differ only slightly more in their degree of genetic relatedness to you and me than do other populations of humans living elsewhere in the world” [54].

So how should the realgattungen *Homo sapiens* be identified if not merely (that is, only distinguishable, from other animals) as a definable biological taxon? Do we have to scientifically define ourselves? Clearly, and as Clark asserts, we humans must “distinguish arten (morphospecies), realgattungen (breeding stock) and metaphysical, regulative forms” [58]; in essence, the ‘constructed reality’ of the scientific, political and religious worldviews of Western society, from reality.

It is the contention of this paper that that reality lies beyond the constructed borders of Western society, and is more exactly relayed through other spheres, such as may be the case as expressed through the narratives of First Peoples, or through the recognition of ‘species’ as fluid phenomenological forms, embedded in what Sterelny terms ‘ecological mosaics’ [61]. It now needs to be embedded back into the Western societal worldview; but does that not mean that reproductively isolated species cannot or should not be recognised?

It is clear that the questions posed of ‘what is an animal?’ and ‘what does it mean to be human?’, of finding ‘our’ place within the ‘animal world’ beyond a constructed reality, are attempts to search for what is supposedly not human in us. In essence, to “characterise the human being stripped of humanity, revealing an animal residue” [45]. One of the many possible consequences of that residue, so aptly put by Ingold, is to shake free from the desire or perceived need to be exploitative of our nonhuman kin, as can be seen across the entire fabric of Western society, and to become socially and ecologically more inclusive of our nonhuman animal kin.
